# Formation of Short-Chain Fatty Acids, Excretion of Anthocyanins, and Microbial Diversity in Rats Fed Blackcurrants, Blackberries, and Raspberries

**DOI:** 10.1155/2013/202534

**Published:** 2013-06-24

**Authors:** Greta Jakobsdottir, Narda Blanco, Jie Xu, Siv Ahrné, Göran Molin, Olov Sterner, Margareta Nyman

**Affiliations:** ^1^Applied Nutrition and Food Chemistry, Department of Food Technology, Engineering and Nutrition, Kemicentrum, Lund University, P.O. Box 124, 221 00 Lund, Sweden; ^2^Centre for Analysis and Synthesis, Department of Chemistry, Kemicentrum, Lund University, P.O. Box 124, 221 00 Lund, Sweden

## Abstract

*Introduction*. Berries contain high amounts of dietary fibre and flavonoids and have been associated with improved metabolic health. The mechanisms are not clear but the formation of SCFAs, especially propionic and butyric acids, could be important. The potent antioxidant and antimicrobial properties of flavonoids could also be a factor, but little is known about their fate in the gastrointestinal tract. *Aim*. To compare how blackcurrants, blackberries, raspberries, and *Lactobacillus plantarum* HEAL19 affect formation of SCFAs, inflammatory status, caecal microbial diversity, and flavonoids. *Results and Conclusions*. Degradation of the dietary fibre, formation of SCFAs including propionic and butyric acids, the weight of the caecal content and tissue, and the faecal wet and dry weight were all higher in rats fed blackcurrants rather than blackberries or raspberries. However, the microbial diversity of the gut microbiota was higher in rats fed raspberries. The high content of soluble fibre in blackcurrants and the high proportion of mannose-containing polymers might explain these effects. Anthocyanins could only be detected in urine of rats fed blackcurrants, and the excretion was lower with HEAL19. No anthocyanins or anthocyanidins were detected in caecal content or blood. This may indicate uptake in the stomach or small intestine.

## 1. Introduction

Berries contain many bioactive compounds such as dietary fibre, phenolic acids, and flavonoids, which may have a number of nutritional properties [1, 2]. Berries and their flavonoids have received much attention in recent years, but little is known about the fate of the flavonoids in the body. Furthermore, few studies have examined dietary fibre in berries. 

Phenolic acids and flavonoids are phytochemicals common in berries, fruits, and vegetables that are a part of a daily diet and may be among their health-promoting factors [2]. Such compounds are also found in, for example, wine and tea. More than 4000 different flavonoids have been described. The dominating group of flavonoids in red berries is anthocyanins, which also predominate in blackcurrants and blueberries [3]. The anthocyanins belong to the flavonoid family and are responsible for the red, orange, and blue colours in fruits [4]. In epidemiological and clinical studies, berries have been associated with improved cardiovascular risk profiles [5] and high consumption of fruits and flavonoids is associated with reduced risk of cardiovascular diseases [3], cancer, coronary heart disease, stroke, and various chronic diseases [2, 6]. In addition to being potent antioxidants, it has also been suggested that they have antimicrobial properties [3, 7] and therefore may play a part in changing the composition of the colonic microbiota [3]. Little is known about microbial degradation, metabolism, and absorption of different flavonoids *in vivo*.

Dietary fibres are not digested in the small intestine and reach the colon intact. In the colon, some of the dietary fibre will become available for fermentation [8, 9]. Dietary fibre in berries and vegetables consists of pectin, xyloglucans, and cellulose [10], which are generally highly fermented. Microbial fermentation yields short-chain fatty acids (SCFAs), gases (CO_2_, CH_4_, and H_2_), and heat. The main SCFAs produced are acetic, propionic, and butyric acids, approximately in the ratio 60 : 20 : 20 [11–13]. However, the colonic degradation and the formation of SCFAs vary, depending on the dietary fibre source, composition of the microbiota, and perhaps the presence of probiotics [14]. SCFAs, and particularly butyrate, are important energy sources for the colonocytes [15]. Consumption of dietary fibre provides many health benefits and some types can reduce the risk of type 2 diabetes, hypertension and obesity [8]. Intake of dietary fibre also decreases the level of serum lipids and promotes weight loss [8]. The SCFAs themselves have also been shown to have positive health effects [16], especially butyric acid which has been shown to be anti-inflammatory [17], to modulate oxidative stress [18], to affect the composition of the mucus layer [19], and to be anticarcinogenic [18, 20]. Some colonic diseases, such as ulcerative colitis and colorectal cancer, have been linked to impaired butyrate metabolism [15, 21]. Propionic acid has been linked to similar effects, but generally to a lesser extent [16, 20], while acetic acid is associated with fewer physiological effects [16]. Dietary fibres are thought to have a preventive role and increased intake of dietary fibres has been linked to a reduction of the above diseases [22, 23]. Poorly fermented fibres may also protect the colonic mucosa by diluting carcinogens and decreasing the transit time, reducing contact time between the carcinogens and the colonic mucosa [24]. Not much is known about fibre-associated antioxidants, whether they are degraded in the colon and whether they affect the formation of SCFAs.

*L. plantarum *HEAL19 is a strain with probiotic potential, and the species *L. plantarum* is spontaneously multiplied to high levels in lactic acid fermented foods of plant origin [25]. *L. plantarum* HEAL19 has pronounced tannase activity and another characteristic is its ability to attach to human mucosa cells *in vitro *[26]. In a study by Osman et al., *L. plantarum* HEAL19, administered separately or with blueberry husks, was shown to reduce the severity of dextran sulphate sodium-(DSS-) induced colitis in rats when compared with the control group fed standard rat chow. It also reduced the levels of the inflammatory marker myeloperoxidase (MPO) [27]. In healthy human volunteers, the phagocytic activity of granulocytes towards *Escherichia coli* was increased following a daily intake of *L. plantarum* HEAL19 for two weeks [28].

There is growing interest in new food components and food supplements with prebiotic properties. Blackcurrants, blackberries, and raspberries are popular berries in Sweden and were therefore selected for this study. The aim was to investigate how these berries, alone or supplemented with *L. plantarum* HEAL19, would affect colonic fermentation in rats. For this purpose, the formation of SCFAs in the hindgut, the microbial diversity in the caecum and the amount of anthocyanins and anthocyanidins in blood, urine, and caecal content were analysed. The body weight gain and the level of SCFAs in portal blood of rats were also measured.

## 2. Materials and Methods

### 2.1. Materials

Blackcurrant (*Ribes nigrum*), blackberry (*Rubus *subgenus *Rubus*), and raspberry (*Rubus ideaus*) were freeze-dried (Probi AB, Lund, Sweden) before addition to rat diets. The bacterial strain used was *Lactobacillus plantarum* HEAL19 (HEAL19, Probi AB, Lund Sweden; DSM 15313). This was administered freeze-dried and mixed daily with the feed at feeding time, at a dose of 10^10^ cfu (colony forming units) per day.

### 2.2. Animals and Diets

Male Wistar rats (Scanbur AB, Sollentuna, Sweden), with an initial weight of 102 (SE 0.8) g, were randomly divided into six groups of seven. The rats were housed individually in metabolic cages [29] in a room maintained at 22°C, with a 12 h light-dark cycle. The feed intake was limited to 12 g (dry weight basis, dwb) per day and water was given *ad libitum*. The protocol for the animal experiment was approved by the Ethics Committee for Animals Studies at Lund University (application number: M 40-06). All diets were adjusted to contain 80 g/kg diet (dwb) of total dietary fibre. Wheat starch was used to increase the carbohydrate concentration. The wheat starch used has been shown to be completely digested and therefore does not form any SCFAs [30]. The rats were fed the berry diets according to Table 1, alone or supplemented with HEAL19. All the rats appeared healthy and active throughout the experimental period and the diets were well tolerated.

The total dietary fibre content (dwb) was 21.7 g per 100 g in blackcurrant, 31.5 g per 100 g in blackberry, and 25.6 g per 100 g in raspberry. Of the dietary fibre content, soluble fibre was 43% in blackcurrant, 18% in blackberry, and 25% in raspberry (Table 2). The polysaccharide composition is shown in Table 2. Dietary fibre in blackberries and raspberries consisted mainly of polymers containing glucose (38%–40%), uronic acids (29%–31%), and xylose (16%–20%). The composition of dietary fibre in blackcurrants was somewhat different, with high content of uronic acids (47%) and mannose (16%), which were very low in the other berries. On the other hand, glucose content (21%) was lower than in the other two berries. All berries contained small amounts of galactose, arabinose, fucose and rhamnose (≤5%), and Klason lignin (5–7 g/100 g, dwb). High content of mannose in blackcurrant has also been reported previously by Hilz et al. [31].

The animals were adapted to the diets for 7 days, followed by a 5-day experimental period, when faeces and feed residues were collected quantitatively, while urine was collected during approximately 1 hour every day. The faecal samples were stored at −20°C and then freeze-dried and milled, before analysis of dietary fibre composition. The urine samples were stored at −20°C until analysis for anthocyanins and anthocyanidins. The feed residues were weighed to calculate the exact amount of ingested feed. After the experimental period, the animals were anaesthetized by subcutaneous injection of a mixture (1 : 1 : 2) of Hypnorm (Division of Janssen-Cilag Ltd., Janssen Pharmaceutica, Beerse, Belgium), Dormicum (F. Hoffmann-La Roche AG, Basel, Switzerland), and water, at a dose of 0.15 mL/100 g body weight. The animals had been fasting for 3–6 h before sacrifice. Blood samples were collected from the portal vein and placed in a serum tube (SST Advance, Plus Blood Collection Tubes, BD, Plymouth, UK), centrifuged, and stored at −40°C until the analysis of SCFAs [32], cytokines, anthocyanins, and anthocyanidins. The caecum was removed and weighed with and without its content, and the pH of the content was measured, before being stored at −40°C for analysis of SCFAs, anthocyanins, and anthocyanidins. Some of the caecal content was weighed and collected in sterile tubes containing freezing medium (water, glycerol (98%), MgSO_4_-7H_2_O, Na-Citrate, KH_2_PO_4_, and K_2_HPO_4_) and immediately frozen in liquid nitrogen for analysis of microbiota diversity. Stool samples were collected from the proximal and distal colon and stored at −40°C until the analysis of SCFAs.

### 2.3. Analytical Methods

#### 2.3.1. Dietary Fibre

The amount of soluble and insoluble dietary fibre in blackcurrant, blackberry, and raspberry was determined at AnalyCen (Skara, Sweden). The method used to analyse the total dietary fibre was AOAC 45.4.07/NMKL 129, the method for insoluble dietary fibre was AOAC 32.1.16, and the soluble dietary fibre was the difference between the two. Soluble and insoluble dietary fibre monomers in the raw material and faeces were characterized using GLC for analysis of neutral sugars and spectrophotometry for analysis of uronic acids [33]. Faecal analysis was performed using the same method but without any prior isolation of dietary fibre. 

#### 2.3.2. SCFAs in Portal Blood

Water and 2-ethylbutyric acid (internal standard) were added to the serum samples, and the SCFAs were protonated with hydrochloric acid. A hollow fibre, for supported liquid membrane extraction, was immersed in the serum solution to extract the SCFAs. After extraction, the SCFAs were flushed from the hollow fibre lumen before being injected onto a fused-silica capillary column (DB-FFAP 125–3237; J&W Scientific, Agilent Technologies Inc., Folsom, CA, USA). GC ChemStation software (Agilent Technologies Inc., Wilmington, DE, USA) was used for the analysis [32].

#### 2.3.3. SCFAs and MCFA in Intestinal Content

The SCFA (acetic, propionic, isobutyric, butyric, isovaleric, valeric, and caproic acids) and one MCFA (medium chain fatty acid, heptanoic acid) in the intestinal content (caecum, proximal and distal colon) were analysed using a GLC method [34]. The intestinal content was homogenized with an Ultra Turrax T25 basic (IKA-Werke, Staufen, Germany) for 1 min after adding hydrochloric acid, to protonate the SCFAs, and 2-ethylbutyric acid (internal standard). The samples were then centrifuged (MSE Super Minor, Hugo Tillquist AB, Solna, Sweden) and the supernatant was analysed as described above. 

#### 2.3.4. Multiple Cytokine Assay

A Milliplex microbeads array system was used to simultaneously measure serum levels of the following 7 cytokines: interleukin (IL)-1*α*, IL-1*β*, IL-6, IL-10, MCP-1, IFN-*γ*, and TNF-*α*. The assay was conducted according to the manufacturer's instructions, using Milliplex MAP rat cytokine kit assay technology. The antibody specific to each cytokine was coupled to microspheres, which were uniquely labelled with a fluorescent dye. The microspheres were incubated with standards, controls, and samples in a 96-well filter plate overnight at 4°C. After incubation, the plate was washed to remove excess reagent, and detection antibodies, one for each of the seven cytokines, were added to the vials. After 2 h incubation at room temperature, streptavidin-phycoerythrin was added for a further 30 min. After a final wash step, the beads were resuspended in buffer and examined with the Luminex 200 instrument (Luminex Corporation, USA) to determine the level of the cytokine of interest. All specimens received were tested in replicate wells. Milliplex Analyst v. 3.4 (Millipore) was used for the evaluation of the results.

#### 2.3.5. Anthocyanins and Anthocyanidins

Standard and calibration solutions were prepared, using cyanidin-3-O-glucoside, delphinidin-3-O-rutinoside (anthocyanins), cyanidin, and delphinidin (anthocyanidins) (Polyphenols Laboratories AS, Sandnes, Norway) at concentrations from 0.5 to 5 *μ*g/mL in 2 M HCl in methanol. For analysis of cyanidin-3-O-sophoroside, delphinidin-3-O-glucoside, and cyanidin-3-O-rutinoside (anthocyanins), the reference values of the wavelength used were those published by Määttä et al. and Ogawa et al. [35, 36]. Anthocyanins and anthocyanidins were extracted from the three berries (See Supplementary Figures  1 and 2 in Supplementry Material available online at http://dx.doi.org/10.1155/2013/202534) and analysed for the above mentioned molecules, on LC-DAD (Waters Alliance Separation Module 2695 fitted with photodiode array detector of Waters, Model 2996) and LC-ESI-MS (Waters Alliance Module 2695 equipped with a 2487 model UV detector). The LC-DAD detected fewer peaks, but those detected were the same as those detected by the LC-ESI-MS. The system was coupled to a Micromass Q-Tof micro (from Waters; assisted with an ESI source in a positive ion electrospray ionization mode).

The content of anthocyanins and anthocyanidins in the samples was analysed with the following methods: urine: 250 *μ*L of urine was mixed with 1 mL MeOH containing 0.05% 2 M HCl and filtrated using a 0.2 *μ*m GHP membrane; caecal content: 0.1 g of caecal content was dissolved in 1 mL MeOH containing 0.05% 2 M HCl and centrifuged; serum: 75 *μ*L of serum and 25 *μ*L of EDTA was mixed with 1 mL MeOH containing 0.05% 2 M HCl and filtrated using a 0.2 *μ*m GHP membrane.

#### 2.3.6. Microbial Diversity

Microbial diversity was analysed using terminal restriction fragment length polymorphism (T-RFLP) [37]. The procedure is listed in the supplementary material.

### 2.4. Calculations and Statistical Analysis

The design of the experiment was randomized. Of the six test diets, three contained berries and three were supplemented with HEAL19. Two-way ANOVA was used to determine the effects of different berries (Berry), HEAL19 (Pro), and their interactions (Berry × Pro). If effects of the different berries (Berry) were significant, the means of the two groups fed the same berry were compared using Tukey's multiple range test (*P* < 0.05). Pearson correlation was used in the correlation evaluations. All evaluations were performed with Minitab software (release 16) with the exception of the Shannon's diversity index H, which was calculated in R. The results were normally distributed with the exception of the T-RFLP analysis, which became normally distributed after Box-Coxing the dataset. Values are presented as means and SEM in Tables 3–6, and differences with *P* values <0.05 are considered significant.

The level of each fatty acid (*μ*mol/g) was multiplied with the caecal amount to obtain the caecal pool (*μ*mol). The body weight gain was calculated in gram per gram food consumed. The faecal excretion (Table 2) of the dietary fibre components was calculated as follows:

(1) $$\begin{array}{c} \frac{{\text{g dietary fibre components in faeces}}_{\text{test diet}}}{{\text{g dietary fibre components ingested}}_{\text{test diet}}}\times 100. \end{array}$$

The fermentability (Table 3) of the dietary fibre was calculated as follows:

(2) $$\begin{array}{c} \left(1-\frac{{\text{g dietary fibre in faeces}}_{\text{test diet}}}{{\text{g dietary fibre ingested}}_{\text{test diet}}}\right)\times 100. \end{array}$$

## 3. Results

### 3.1. Feed Intake and Body Weight

The feed intake in the blackberry groups was lower than in the blackcurrant and raspberry groups (11.2 g versus 12.0 ± 0.01 g, resp., *P* < 0.001) and the dietary fibre intake was also lower (4.5 g versus 4.8 ± 0.004 g, *P* < 0.001) (Table 3). No effect on feed and dietary fibre intake of HEAL19 could be seen for any of the diets. The weight gain for rats fed blackberry and HEAL19 was lower (0.13 g/g feed) than for rats fed blackcurrant and HEAL19 (0.24 g/g feed, *P* = 0.006). No other differences in weight gain were registered.

### 3.2. Caecal Content, Caecal Tissue, Caecal pH, Dietary Fibre Fermentability, and Faecal Weights

The amount of caecal content was similar for the blackberry and raspberry groups, (1.9 ± 0.06 g), while the content was higher (2.5 g, *P* < 0.001) in the blackcurrant groups (Table 3). The caecal tissue weight was the lowest in the blackberry groups (0.42 g), followed by the raspberry groups (0.47 g), but again the highest in the blackcurrant groups (0.71 g, *P* = 0.003). No differences were detected in the caecal pH of the rats.

The faecal wet weight was higher in the blackcurrant groups (14.6 g) than in the blackberry (9.1 g) and raspberry groups (10.1 g, *P* < 0.05). However, when considering the faecal dry weight, the only relevant significance was between the group fed blackcurrant (7.2 g) and the raspberry group (5.7 g, *P* < 0.01). The fermentability of the dietary fibre was higher for the blackcurrant groups (75.0%) than for the raspberry (62.5%) and blackberry (63.5%) groups (*P* < 0.001). The dietary fibre residues in faeces were lower in the blackcurrant groups (1.2 g) than for the blackberry and raspberry groups (1.7 ± 0.04 g, *P* < 0.001). The addition of HEAL19 had no effect on the results reported above. 

Dietary fibre polymers containing uronic acids were more fermented in all groups, compared with the other dietary fibre monomers, and only 11% was excreted in the faeces of the blackcurrant groups and 15 ± 0.6% in the two other groups (Table 2). Of the other main components in the dietary fibre, xylose and glucose were also more fermented by the blackcurrant groups. The difference in fermentability can most probably be explained by the higher content of soluble fibres in blackcurrant than in raspberry and blackberry (Table 2).

### 3.3. SCFAs in Intestinal Content

**Figure 1:**
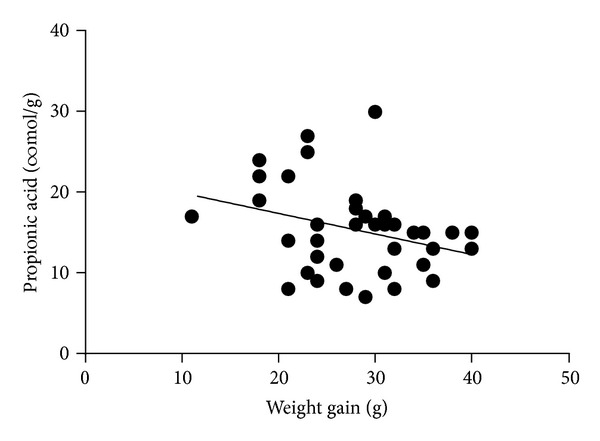
The level of propionic acid decreased with weight gain (*P* < 0.05, correlation factor −0.317).

No significant differences in the levels of SCFAs in caecal content were seen between any of the groups, even though the levels of propionic acid and minor acids (isobutyric, valeric, isovaleric, caproic and heptanoic acids) were close to significance (*P* = 0.069 and *P* = 0.057, resp.) (data not shown). On the other hand, the total caecal pool of SCFAs was higher in rats fed blackcurrants than the other berries (152 versus 105 ± 5.4 *μ*mol, *P* = 0.002) (Table 4). The pool of the individual acids, except the minor acids, was also higher with blackcurrants. The caecal pool of acetic acid in the blackcurrant groups was 109 *μ*mol versus 74 ± 4.0 *μ*mol for rats fed the other two berries (*P* = 0.002). For propionic acid, the caecal pool was 20 *μ*mol for the blackcurrant groups versus 13 ± 0.6 *μ*mol for the other berries (*P* < 0.001) and for butyric acid it was 17 *μ*mol versus 13 ± 0.7 *μ*mol for the blackcurrant group and the other berries, respectively (*P* = 0.032). No effect of HEAL19 could be seen. In addition, the level of propionic acid was negatively correlated with weight gain (*P* < 0.05) (Figure 1).

Similar results were seen for SCFAs in the proximal and distal colon (Table 4), with blackcurrant generally generating higher levels than the other groups. The proximal level of acetic acid was 31 *μ*mol/g versus 21 ± 1.9 (*P* < 0.001) for the other groups. Furthermore, the level of propionic acid was 5.4 *μ*mol/g in rats fed blackcurrant versus 3.9 *μ*mol/g (*P* = 0.005) in rats fed blackberry. Butyric acid was higher in rats fed raspberry than blackberry or blackcurrant (4.8 versus 3.5 *μ*mol/g, *P* = 0.038). In the distal colon, both blackcurrant and raspberry generally seemed to give higher levels in the rats than blackberry. In rats fed blackcurrant together with HEAL19, the total SCFAs and acetic acid in the distal colon were greater than without HEAL19 (*P* < 0.05). The levels of propionic and butyric acids were higher in the blackcurrant groups compared with the blackberry groups (*P* < 0.001 and *P* = 0.041, resp.).

The relative mean distribution of acetic, propionic, and butyric acids of all groups was as follows: in the caecum, acetic acid 74%, propionic acid 13%, and butyric acid 13%; in proximal colon, acetic acid 75%, propionic acid 13%, and butyric acid 12%; in distal colon, acetic acid 71%, propionic acid 14%, and butyric acid 15%.

### 3.4. SCFAs in Portal Blood

Acetic acid was the major acid detected in the portal serum for all groups (734–1050 *μ*mol/L), followed by propionic (54–75 *μ*mol/L) and butyric acids (48–55 *μ*mol/L) (Table 5). Considerable amounts of isobutyric (10–12 *μ*mol/L), isovaleric (9–12 *μ*mol/L), and valeric acids (4-5 *μ*mol/L) were also formed. Rats fed blackcurrants had the highest level of acetic acid (*P* = 0.007) and the total amount of SCFAs (*P* = 0.016). For all groups, the relative mean distribution of acids was 89% (acetic acid), 6% (propionic acid), and 5% (butyric acid). Addition of HEAL19 had no effect.

### 3.5. Inflammatory Markers

**Figure 2:**
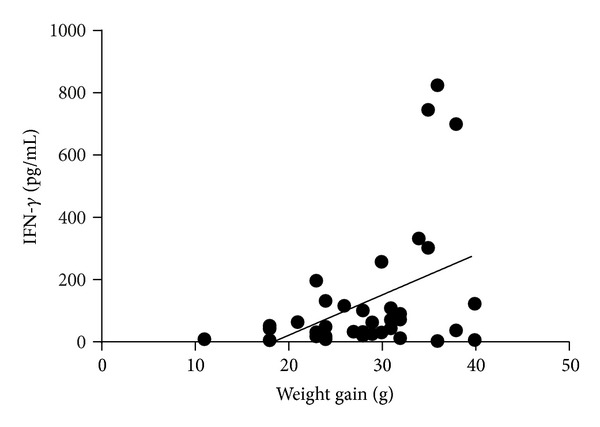
The level of the inflammatory cytokine IFN-*γ* increased with weight gain (*P* < 0.01, correlation factor 0.422).

**Figure 3:**
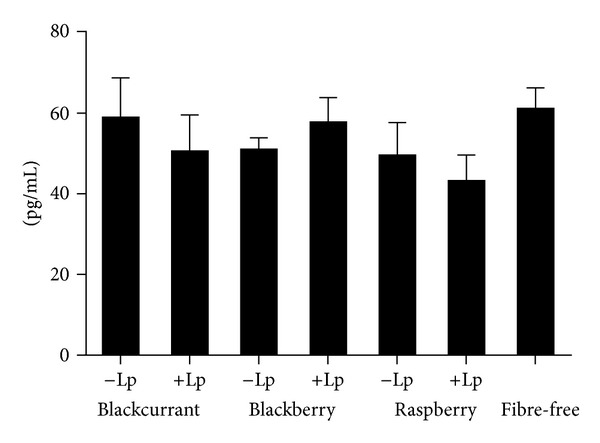
Level (pg/mL) of MCP-1 in rats fed diets containing blackcurrant, blackberry, and raspberry, with the same diets supplemented with *L. plantarum* HEAL19, and a fibre-free group from another study.

The inflammatory cytokine IFN-*γ* was positively correlated with weight gain (*P* < 0.01) (Figure 2). No difference was seen in rats fed the different berries for the individual cytokines, INF-*γ*, MCP-1, and IL-1*α*, but the raspberry group together with HEAL19 tended towards a lower level of MCP-1, especially when compared with a fibre-free group from another study (*P* = 0.07) (Figure 3). The levels of the other four cytokines (IL-1*β*, IL-6, IL-10, and TNF-*α*) were below the detection limit (i.e., <2.2, 16.9, 10.4, and 4.4 pg/mL, resp.).

### 3.6. Anthocyanins and Anthocyanidins

The blackberries gave most peaks, followed by raspberries and blackcurrants (Table 6). Delphinidin and cyanidin (anthocyanidins) were only detected in blackberries, while cyanidin-3-O-sophoroside was only found in raspberries. Blackcurrants and blackberries contained high amounts of cyanidin-3-O-rutinoside and delphinidin-O-3-rutinoside and, to some extent, cyanidin-3-O-glucoside, but those compounds were lower in raspberries. 

None of the compounds analysed in the berries were detected in the portal serum or in the caecal content. However, anthocyanins were detected in the urine of rats fed blackcurrant, but not in the other groups. Rats fed blackcurrant alone had higher levels of anthocyanins in urine than those supplemented with HEAL19 (61 versus 29.8 *μ*g/mL (*P* = 0.029) for delphinidin-3-O-rutinoside, 54.9 versus 26.8 *μ*g/mL (*P* = 0.056) for cyanidin-3-O-rutinoside, 32.8 versus 23.2 *μ*g/mL (*P* = 0.11) for delphinidin-3-O-glucoside, and 170.5 versus 96.6 *μ*g/mL (*P* = 0.045) for the total amount excreted) (Table 6).

### 3.7. T-RFLP Analysis

**Figure 4:**
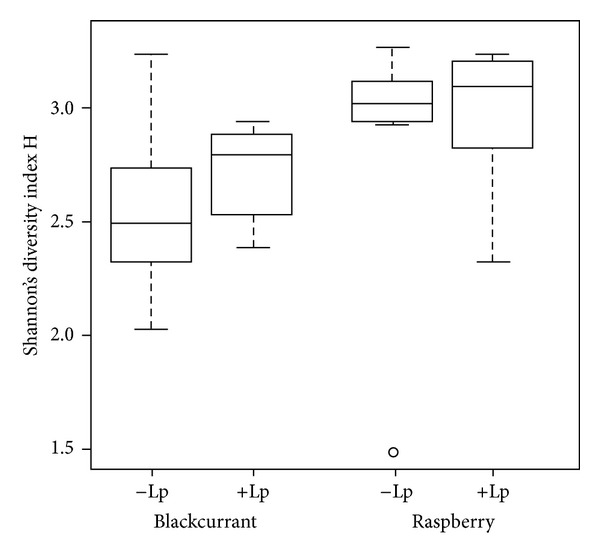
Shannon's diversity index H of the caecal microflora of rats fed blackcurrant and raspberry, with the same diets supplemented with *L. plantarum* HEAL19.

The 16S rRNA gene was successfully amplified from the caecal content of the rats fed raspberries and blackcurrants, but failed for the rats fed blackberries. (The reason for this is unknown, but the high concentration of glycerol in the freezing media might have worked as an inhibitor in the PCR reaction. Dilution and other attempts of PCR optimization did not work.) There was a clear difference in diversity index and relative abundance of certain T-RFs, representing bacterial genus or species, between the rats fed blackcurrants and raspberries. The diversity index was higher for the rats fed raspberries than blackcurrants (2.9 versus 2.6, *P* = 0.026) (Figure 4). The addition of HEAL19 did not result in any significant change of microbial diversity in the two groups, even though the median values for the groups with added HEAL19 were slightly higher. PCA analysis of the caecal microbiota in rats fed blackcurrants and raspberries showed difference in the compositions; PC1 explained 36.1% of the variance and PC2 19.3%. Larger individual variations in the microbiota composition were seen in rats fed blackcurrants, while relatively homogeneous microbiota were shared by the rats in the raspberry group. Certain bacterial species were highly represented in the blackcurrant groups (i.e., T-RF93.17) and in the raspberry groups (i.e., T-RF467.98). The *Lactobacillus plantarum* HEAL19 should have a position at T-RF568, but it could not be detected in any of the samples.

## 4. Discussion

The present study demonstrates that dietary fibre, and perhaps anthocyanins, in blackcurrants is more available to the hindgut microflora in conventional rats than the corresponding components in blackberries and raspberries, and this may have implications on metabolic and gastrointestinal health. Groups fed blackcurrants degraded the dietary fibre to a greater extent and had higher levels of SCFAs in the hindgut and also heavier caecal content and tissue than the other groups. On the other hand, the microbial diversity was lower in groups fed blackcurrants than those fed raspberries. Furthermore, the anthocyanins measured in blackcurrants could be detected in the urine of rats, but not in those fed blackberries and raspberries. All the above results are probably associated with the higher proportion of soluble fibre in blackcurrant than in the other two berries, which is also reflected in the considerably lower proportion of cellulose (glucose). The high content of polymers composed of mannose may also have an effect.

Blackcurrant resulted in the highest level of propionic acid in the caecum, proximal, and distal colon, which might be due to the higher content of polymers containing mannose in blackcurrants compared with the other two berries. Interestingly, guar gum, a polysaccharide consisting of galactomannan, has been shown to give rise to high levels of propionic acid [38]. Guar gum *per se* has been associated with both lowered cholesterol levels [39] and lower postprandial blood glucose levels [40], but no investigation was made of whether this was associated with the viscosity of guar gum or its capacity to form high amounts of propionic acid. The blackcurrant also resulted in higher levels of butyric acid in the caecum and distal colon of rats. Indigestible dietary fibre components, such as *β*-glucans and resistant starch, have also been shown to give rise to high amounts of butyric acid [41–43]. This is sometimes explained by the fact that these fibres consist of soluble polymers containing glucose [42, 44]. The glucose in blackcurrants was also fermented to higher extent (60%) than in the other two berries (approximately 50%), indicating a higher solubility of the polymeric glucose in blackcurrants. Similarly, oat *β*-glucans of high solubility have been shown to give rise to higher levels of butyric acid when compared with cellulose [42]. The importance of the formation of high amounts of SCFAs for colonic health, particularly butyric and propionic acids, in the distal part of the colon has been reported previously [16, 20]. Propionic and especially butyric acids are important substrates for the colonic epithelial cells and improve gut health [20].

Minor effects were seen when the probiotic strain HEAL19 was added. The reason is not known but may be due to the experiment being performed using conventional rats with a high load of resident *Lactobacillus *well adapted to the gastrointestinal tract of rats, or that the experiment was quite short in terms of time. Perhaps it may also be difficult to affect healthy animals. However, with blackcurrants, there was an increased level of acetic acid in distal colon, and the same was seen in blood. Similar results have been observed with blueberry husk and multistrain probiotics [14]. The *Lactobacillus plantarum* species are known to ferment pentoses and/or gluconate to lactic and acetic acids [26]. It might therefore be speculated whether the addition of HEAL19 to blackcurrant increases formation of acetic acid. However, it is more probable that the HEAL19 has interacted with the resident microbiota in a way that enhanced the acetic acid producing capacity.

Dietary fibres in blackberries and raspberries were quite resistant to microbial colonic degradation, as 40% of the dietary fibre eaten was excreted in the faeces. The amount of caecal content and the weight of the caecal tissue was the highest in the blackcurrant groups, which may be due to the higher degree of fermentation of these types of dietary fibre and consequently greater formation of SCFAs. In this study, there was a correlation between the caecal tissue weight and the caecal content (correlation factor 0.69, *P* < 0.001), while the correlation of the caecal tissue weight and content to SCFA levels was 0.54 and 0.85, respectively (*P* < 0.001). Studies in the literature are not consistent on this matter, and the increased caecal tissue weight is sometimes related to increased caecal levels of SCFAs and sometimes to the caecal content [14, 45, 46].

Blackcurrants and raspberries resulted in clear differences in the caecal microbiota. Raspberries resulted in higher microbial diversity in caecum than blackcurrants. Adding HEAL19 to the diet had only minor effects. The treatment time (12 d) is probably too short to significantly increase/affect the microbial diversity, in addition to the fact that the rats in the experiment were healthy and probably with a high load of resident *Lactobacillus*. Compared with a fibre-free, high-fat diet, the diversity was greater with the berries (2.6 and 2.9 versus 2.0) (G. Jakobsdottir, J. Xu, S. Ahrné, G. Molin, and M. E. Nyman, unpublished results). Similar effects have been seen in obese individuals; when the diversity of the faecal microbiota was compared with their lean twins, the diversity was lower in the obese twins [47, 48]. Despite the lower microbial diversity in rats fed blackcurrants, the results were surprising because the dietary fibre in blackcurrant was more fermented and resulted in high levels of SCFAs. It may therefore be speculated that less diversity in rats fed blackcurrant is because dietary fibre is more soluble and easily fermented, a situation that favours fast-growing microbes and results in lower number of bacterial species needed to metabolise/ferment the dietary fibre. On the other hand, for raspberries, which contain more insoluble and less fermentable dietary fibre, different bacterial species are required for the degradation, thereby increasing diversity. Interestingly, in spite of generally lower diversity in rats fed blackcurrant, they showed larger individual variations in the microbiota composition. A reasonable explanation for this is that blackcurrant contains more easily fermentable dietary fibre and so allows the development of several fast-growing or opportunistic microbes, which may be different in each animal. The fate of HEAL19 could not be determined and, in most cases, no differences were seen, with or without it. However, the group fed blackcurrant and HEAL19 formed higher levels of acetic acid and showed lower anthocyanin excretion, compared with the case when HEAL19 was not added. It may therefore be speculated that one or few species of the microbial flora increased and that this/these species produced greater amounts of acetic acid. In most cases, however, HEAL19 was either excluded by existing microflora, or it may have needed longer time to establish itself in the gut. In another study on C57BL/6J mice fed a high-fat diet with green tea powder and HEAL19 for 22 weeks, HEAL19 was detectable in the gut  [37]. HEAL19 was also detectable in DSS treated rats fed blueberries and HEAL19 for 6 months [49].

*Lactobacillus plantarum *can degrade tannins [26, 50], but tannins and other polyphenols are also known to have antimicrobial properties [51]. Raspberries contain large amounts of hydrolysable tannins [52]. The phenolic proportion of hydrolysable tannins is approximately 85% in raspberries [53–55], while blackcurrants only contain approximately 10% proanthocyanidins and no hydrolysable tannins [54]. Tannins affect bacterial growth in several ways, for example, by inhibiting extracellular enzymes, or by forming complexes with metal ions, proteins, and polysaccharides needed for growth [56, 57]. A study on blueberry husk showed antimicrobial effect of the blueberries, which might be due to the high levels of phenolic compounds and tannins [14]. This might explain why the formation of SCFAs is stimulated by adding blackcurrants, which contain little or no tannins.

The anthocyanin composition demonstrated in this study for blackcurrant is consistent with previous measurements [54]. However, the anthocyanin and anthocyanidin compositions for raspberry and blackberry were different compared with previous studies, which might be due to use of different cultivars or different analytical methods. The anthocyanins detected in blackcurrants were also detected in the urine, but not in the rats fed blackberries or raspberries. This indicates that the anthocyanins in blackcurrants were absorbed before reaching the colon and some of them were then excreted in the kidneys. The rats fed blackcurrants ate larger amounts of berries, due to the lower amount of dietary fibre. Blackcurrant also contained larger amounts of these components, and both these factors may explain why the anthocyanins were only detected in those groups. Rats fed HEAL19 excreted less delphinidin-3-O-rutinoside and total amount of anthocyanins, indicating that the anthocyanins were metabolised to a greater extent in the rats fed HEAL19. There is no consensus in the literature concerning the uptake and fate of the anthocyanins, anthocyanidins, and tannins in the body. Some authors state that most of them reach the large intestine, where they become substrate for the colonic microflora [51, 58], while others have demonstrated uptake in the stomach and small intestine [59]. It is also important to remember that these compounds have different compositions and properties and may therefore act differently in the body. Flavonoids (anthocyanins and anthocyanidins are a subgroup of flavonoids) have been shown to be excreted to a large degree in urine, but some parts are unabsorbed and excreted in faeces, probably because they are bound to the dietary fibre resistant to colonic fermentation. Some may be liberated from the dietary fibre during microbial degradation, and could even reenter the jejunum with the bile and then be reabsorbed or excreted in faeces [59]. Molecules derived from the anthocyanins and anthocyanidins and found in urine, blood, caecal, or colonic content may be different to those in the raw material due to rapid metabolism and colonic fermentation [51]. Anthocyanins and anthocyanidins could not be detected in the caecal content or serum for any of the groups, which indicates that they are absorbed in the stomach and in the small intestine and that they are not bound to the dietary fibre. Rats whose diet was supplemented with HEAL19 excreted lower amounts in urine than rats without HEAL19, which also indicates that the probiotic strain in some way increased the utilization/metabolization in the upper part of the gut [59]. In such cases, the levels should increase relatively fast after consumption of foods rich in anthocyanins and anthocyanidins. On the other hand, the levels might be relatively low and therefore hard to detect. In this study, the rats were not given any new food before being anaesthetized, which might affect and reduce the levels in blood. The levels of the anthocyanins and anthocyanidins may also have been below the detection limit of the method used, or the extraction may not have been efficient enough, since others have reported anthocyanins and anthocyanidins in caecal and colon content [58]. Metabolites formed by the caecal microflora from the anthocyanins and anthocyanidins may also be absorbed into the circulation but are hard to detect.

Interestingly, a correlation was shown between weight gain and the level of IFN-*γ*. Similar results on overweight 10-11-year-old boys have been reported, where elevated levels of IFN-*γ*, MCP-1, IL-6, IL-8, CRP, and insulin were detected compared with boys of normal weight [60]. No significant differences could be seen between the groups fed the different berries in terms of the other inflammatory markers detected. However, for the levels of MCP-1, a trend towards a reduced level could be seen in rats fed raspberry and HEAL19 compared with the other berries, and especially with a group fed a fibre-free diet in another study (*P* = 0.07). It may be speculated whether this is influenced by the difference in the microbial diversity. Serum MCP-1 levels have been shown to be higher in type 2 diabetes patients compared with healthy controls, and there are also reports that blood glucose levels may increase the levels of MCP-1 [61]. All the groups had IL-1*β* levels below the detection limit, while rats fed a fibre-free diet in another study had higher levels of IL-1*β* (*P* < 0.001).

## 5. Conclusions

In conclusion, a distinct difference was observed for nearly all measured parameters between rats fed blackcurrants and those fed blackberries and raspberries. However, addition of *L. plantarum *HEAL19 did not show any significant effects during this relatively short exposure. Fermentability of the dietary fibre, formation of SCFAs, the levels of SCFAs in hindgut and portal serum, the weight of the caecal content and tissue, and the faecal wet and dry weight were all higher for the rats fed blackcurrants than the other two groups. On the other hand, the microbial diversity was lower than in rats fed raspberries. Anthocyanins were excreted in the urine of rats fed blackcurrants but not in rats fed blackberries and raspberries. No anthocyanins or anthocyanidins were detected in caecum, which may indicate an uptake in the stomach or small intestine, or at least theoretically that these compounds are rapidly metabolised by the microbiota in the caecum and colon. The high content of soluble fibre in blackcurrant, as well as low cellulose (glucose) and possibly also the high proportion of mannose-containing polymers, is probably the main explanation for these results.

## Supplementary Material

Anthocyanins and anthocyanidins were extracted from blackcurrants and blackberries using a three-step extraction; ethyl acetate extraction, methanol extraction and methanol extraction of hydrolysed anthocyanidins. The extraction for raspberries was a two-step extraction; ethyl acetate extraction and methanol extraction.

## Figures and Tables

**Table 1:** Composition of test diets^1^ (g/kg, dwb).

Component	Blackcurrant	Blackberry	Raspberry
Berries^†^	368	253	313
Casein	120	120	120
DL-methionine	1.2	1.2	1.2
Maize oil	50	50	50
Mineral mixture^‡^	48	48	48
Vitamin mixture^$^	8	8	8
Choline chloride	2	2	2
Sucrose	100	100	100
Wheat starch^*∧*^	302.8	417.8	357.8

^1^Half of the berry diets were supplemented with HEAL19 (Lactobacillus plantarum HEAL19) at a dose of 10^10^ cfu per day and mixed daily with the feed at feeding time.

^†^Corresponding to 80 g indigestible carbohydrate/kg diet (dwb), berries obtained from Probi AB (Lund, Sweden).

^‡^Containing (g/kg): 0.43 CuSO_4_ · 5H_2_O, 1.6 ZnSO_4_ · 7H_2_O, 389 KH_2_PO_4_, 201.2 NaH_2_PO_4_ · 2H_2_O, 380 CaCO_3_, 0.08 KI, 67 MgSO_4_, 9 FeSO_4_ · 7H_2_O, 4 MnSO_4_ · H_2_O, 0.023 CoCl · 6H_2_O, 119.1 NaCl, 0.019 chromium (III) chloride, and 0.013 sodium selenate.

^$^Containing (g/kg): 0.62 menadione, 2.5 thiamin hydrochloride, 2.5 riboflavin, 1.25 pyridoxine hydrochloride, 6.256 calcium pantothenate, 6.256 nicotinic acid, 0.25 folic acid, 12.5 inositol, 1.25 p-aminobenzoic acid, 0.05 biotin, 0.00375 cyanocobalamin, 0.187 retinol palmitate, 0.00613 calciferol (D3), 25 d-*α*-tocopheryl acetate, and 941.25 maize starch (Apoteket, Malmö, Sweden).

^*∧*^Norfoods Sweden AB, Malmö, Sweden.

**Table 2:** Content (g/100 g, dwb) and faecal excretion (%) of dietary fibre in rats fed a diet containing blackcurrant, blackberry, and raspberry, and *L. plantarum* HEAL19.

	Content and composition (g/100 g, dwb)							Faecal excretion (% of intake)			
	Blackcurrant		Blackberry		Raspberry		*P*	Blackcurrant	Blackberry	Raspberry	*P*
Rhamnose	0.2^b^	(1)	0.4^a^	(2)	0.3^b^	(2)	0.001	36^b^	41^a^	40^ab^	0.046
Fucose	0.03^b^	(<0.5)	0.1^a^	(<0.5)	0.04^b ^	(<0.5)	0.008	65	59	56	NS
Arabinose	0.8^b^	(5)	1.1^a ^	(4)	1.1^c^	(6)	<0.001	17^b^	16^b^	10^a^	<0.001
Xylose	0.8^b^	(5)	5.1^a ^	(20)	3.1^c^	(16)	<0.001	26^a^	49^b^	54^b^	<0.001
Mannose	2.6^a^	(16)	0.4^b^	(2)	0.4^b^	(2)	<0.001	43	35	33	NS
Galactose	0.9	(5)	0.7	(3)	0.9	(5)	NS	25^b^	41^a^	22^b^	<0.001
Glucose	3.5^b^	(21)	10.2^a^	(40)	7.2^c^	(38)	<0.001	40^a^	51^b^	53^b^	<0.001
Uronic acids	7.9^b^	(47)	7.5^b^	(29)	5.9^a^	(31)	0.011	11^a^	15^b^	16^b^	<0.001
Dietary fibre polysaccharides	16.7^b^	(100)	25.5^a^	(100)	18.9^c^	(100)	<0.001	25^b^	37^a^	38^a^	<0.001
Klason lignin	5.0^a^		6.0^ab^		6.7^b^		0.032	ND	ND	ND	
Total dietary fibre^1^	21.7		31.5		25.6						

Values within parentheses show the composition of dietary fibre (%).

^1^The proportion of soluble dietary fibre was for blackcurrant 43%, blackberry 18%, and raspberry 25%.

^a,b,c^Mean values within a row, with unlike superscripts are significantly different (*P* < 0.05).

**Table 3:** Feed and dietary fibre intake, body weight gain, caecal content, caecal tissue weight, caecal pH, dietary fibre fermentability, and faecal wet and dry weight in rats fed diets containing blackcurrant, blackberry, and raspberry, with the same diets supplemented with *L. plantarum* HEAL19.

	Blackcurrant				Blackberry				Raspberry					*P*	
	−HEAL19		+HEAL19		−HEAL19		+HEAL19		−HEAL19		+HEAL19			
	Mean	SE	Mean	SE	Mean	SE	Mean	SE	Mean	SE	Mean	SE	Berry	Pro	Berry × Pro
Feed intake (g/d)	11.9	0.01	11.9	0.03	11.1	0.3	11.2	0.31	12.0	0.0	12.0	0.0	<0.001^1^	NS	NS
Dietary fibre intake (g/5 d)	4.8	0.0	4.8	0.0	4.5	0.1	4.5	0.1	4.8	0.0	4.8	0.0	<0.001^1^	NS	NS
Body weight gain (g/g feed)	0.17^ab^	0.01	0.24^a^	0.01	0.17^ab^	0.03	0.13^b^	0.03	0.22^a^	0.01	0.18^ab^	0.02	0.018	NS	0.016
Caecal content (g)	2.7	0.15	2.4	0.18	1.8	0.08	1.9	0.11	2.0	0.08	1.9	0.11	<0.001^2^	NS	NS
Caecal tissue weight (g)	0.7	0.02	0.7	0.01	0.4	0.01	0.4	0.01	0.4	0.01	0.5	0.01	<0.001^3^	NS	NS
Caecal pH	7.2	0.08	7.1	0.04	7.2	0.07	7.1	0.07	7.2	0.09	7.0	0.08	NS	NS	NS
Fibre fermentability (%)	72.3	3.0	77.8	2.0	64.4	1.0	61.8	1.0	61.3	1.2	62.5	1.5	<0.001^2^	NS	NS
Faecal wet wt (g/5 d)	16.1^c^	1.2	13.1^b^	0.8	9.0^a^	0.3	9.3^a^	0.4	10.1^a^	0.3	10.2^a^	0.3	<0.001	NS	0.032
Faecal dry wt (g/5 d)	7.2^a^	0.8	6.2^ab^	0.5	6.4^ab^	0.3	7.1^a^	0.3	5.7^b^	0.2	6.3^ab^	0.2	0.032	NS	0.008
Faecal fibre residue (g/5 d)	1.3	0.2	1.1	0.1	1.6	0.06	1.7	0.08	1.8	0.09	1.8	0.1	<0.001^4^	NS	NS

Mean values with their SEM for seven rats per group.

^1^Values for groups fed blackberry were significantly lower than those fed blackcurrant and raspberry.

^2^Values for groups fed blackcurrant were significantly higher than those fed blackberry and raspberry.

^3^Values for groups fed blackcurrant were significantly higher than those fed blackberry and raspberry (*P* < 0.001), and groups fed raspberry were significantly higher than groups fed blackberry (*P* = 0.0044).

^4^Values for groups fed blackcurrant were significantly lower than those fed blackberry and raspberry.

^a,b,c^Mean values within a row, with unlike superscripts are significantly different (*P* < 0.05).

**Table 4:** Short-chain fatty acids in caecum (*µ*mol/caecal content) and proximal and distal colon (*µ*mol/g) of rats fed diets containing blackcurrant, blackberry, and raspberry, with the same diets supplemented with *L. plantarum* HEAL19.

	Blackcurrant				Blackberry				Raspberry					*P*	
	−HEAL19		+HEAL19		−HEAL19		+HEAL19		−HEAL19		+HEAL19			
	Mean	SE	Mean	SE	Mean	SE	Mean	SE	Mean	SE	Mean	SE	Berry	Pro	Berry × Pro
*Caecal pool *															
Acetic	102.9	11.9	114.3	14.6	73.5	8.1	69.9	11.4	80.8	7.4	71.2	6.3	0.002^1^	NS	NS
Propionic	20.4	2.5	19.5	2.1	12.8	1.3	12.3	1.7	13.8	1.0	12.3	1.1	<0.001^1^	NS	NS
Butyric	17.9	1.8	16.7	2.4	13.0	1.5	12.5	2.0	14.2	1.5	13.4	1.1	0.032^2^	NS	NS
Minor	6.4	0.8	6.4	1.2	5.9	0.5	5.2	0.6	5.5	0.6	4.9	0.5	NS	NS	NS
Total (*µ*mol)	**148**	**16**	**157**	**20**	**105**	**11**	**100**	**15**	**114**	**10**	**102**	**9**	**0.0021**	**NS**	**NS**

*Proximal *															
Acetic	31.0	1.6	30.4	2.6	20.4	2.6	21.1	2.9	29.5	2.9	28.9	0.8	<0.001^3^	NS	NS
Propionic	5.5	0.6	5.4	0.7	4.2	0.4	3.6	0.4	4.7	0.4	4.6	0.2	0.005^2^	NS	NS
Butyric	4.5	0.4	3.9	0.5	3.7	0.7	3.3	0.3	4.4	0.4	5.1	0.6	0.038^4^	NS	NS
Minor	1.5	0.1	1.4	0.1	1.5	0.2	1.4	0.2	1.6	0.1	1.5	0.1	NS	NS	NS
Total (*µ*mol/g)	**42**	**2**	**41**	**4**	**30**	**3**	**29**	**3**	**40**	**4**	**40**	**2**	**<0.0013**	**NS**	**NS**

*Distal *															
Acetic	28.7^b^	2.3	40.6^a^	2.6	24.2^b^	3.8	22.9^b^	2.0	28.4^b^	1.5	25.8^b^	1.1	<0.001	NS	0.007
Propionic	5.8	0.7	7.1	0.6	4.8	0.6	4.8	0.3	4.7	0.3	4.5	0.3	0.001^1^	NS	NS
Butyric	6.3	1.1	7.7	0.9	5.0	0.5	5.1	0.9	6.4	0.4	6.3	0.3	0.041^2^	NS	NS
Minor	2.1	0.2	2.7	0.3	2.5	0.2	2.2	0.2	2.6	0.3	2.0	0.1	NS	NS	NS
Total (*µ*mol/g)	**43** ^ b^	**4**	**58** ^ a^	**4**	**36** ^ b^	**5**	**35** ^ b^	**3**	**42** ^ b^	**2**	**39** ^ b^	**1**	**<0.001**	**NS**	**0.021**

Mean values with their SEM for seven rats per group, six rats per group for blackberry +HEAL19 (caecal pool), for blackberry (proximal and distal colon), and for blackcurrant +HEAL19 (proximal colon).

^1^Groups fed blackcurrant were significantly higher than those fed blackberry and raspberry.

^2^Groups fed blackcurrant were significantly higher than those fed blackberry.

^3^Groups fed blackcurrant and raspberry were significantly higher than those fed blackberry.

^4^Groups fed raspberry were significantly higher than those fed blackberry.

^a,b^Mean values within a row, with unlike superscripts are significantly different (*P* < 0.05).

**Table 5:** Levels of short-chain fatty acids (*µ*mol/L) in portal serum of rats fed diets containing blackcurrant, blackberry, and raspberry, with the same diets supplemented with *L. plantarum* HEAL19.

	Blackcurrant				Blackberry				Raspberry					*P*	
	−HEAL19		+HEAL19		−HEAL19		+HEAL19		−HEAL19		+HEAL19			
	Mean	SE	Mean	SE	Mean	SE	Mean	SE	Mean	SE	Mean	SE	Berry	Pro	Berry × Pro
Acetic	931	54	1050	83	862	65	870	77	827	66	734	19	0.007^1^	NS	NS
Propionic	69.5	6.3	74.8	11.9	59.8	6.8	60.2	9.5	55.1	7.5	54.4	4.6	NS	NS	NS
Butyric	54.6	4.6	49.1	8.2	52.1	8.4	48.0	9.4	48.1	6.8	48.7	4.5	NS	NS	NS
Isobutyric	10.3	0.8	11.3	1.8	12.4	0.6	12.0	0.9	11.2	1.5	10.7	0.4	NS	NS	NS
Isovaleric	8.9	0.6	9.9	1.9	11.1	0.8	12.1	1.4	11.6	2.0	9.9	0.6	NS	NS	NS
Valeric	4.4	0.5	4.7	1.5	4.8	0.7	4.8	1.3	4.0	0.7	3.9	0.7	NS	NS	NS

Total	1079	63	1200.0	101.0	1002	76	1007	91	957	80	862	26	0.016^1^	NS	NS

Mean values with their SEM for seven rats per group.

^1^Groups fed blackcurrant were significantly higher than those fed raspberry.

**Table 6:** Anthocyanins and anthocyanidins detected with LC-ESI-MS from blackcurrant, blackberry, and raspberry (mg/g, dwb), with amount consumed (mg/5 days) and the amount excreted in urine of rats fed blackcurrant (*µ*g/mL).

Anthocyanins and anthocyanidins	Amount in berries (mg/g, dwb)			Amount consumed (mg/5 day)						Amount excreted-Blackcurrant (*µ*g/mL)			
	Blackcurrant	Blackberry	Raspberry	Blackcurrant		Blackberry		Raspberry		−HEAL19		+HEAL19	
	Mean	Mean	Mean	Mean	SE	Mean	SE	Mean	SE	Mean	SE	Mean	SE
Cyanidin-3-O-shoporoside	ND	ND	0.80	—	—	—	—	15.0	0.0	—	—	—	—
Delphinidin-3-O-glucoside	0.73	0.6	0.80	16.1	0.02	8.5	0.16	15.0	0.0	32.8	3.4	23.2	3.8
Delphinidin-3-O-rutinoside	1.71	1.35	0.39	37.6	0.06	19.2	0.36	7.3	0.0	61.0	9.8	29.8*	4.9
Cyanidin-3-O-glucoside	0.34	0.44	0.14	7.5	0.01	6.2	0.12	2.6	0.0	21.9	3.7	16.8	1.7
Cyanidin-3-O-rutinoside	1.49	1.3	0.19	32.8	0.05	18.4	0.34	3.6	0.0	54.9	11.2	26.8	4.0
Delphinidin	ND	0.23	ND	—	—	3.3	0.06	—	—	—	—	—	—
Cyanidin	ND	0.15	ND	—	—	2.1	0.04	—	—	—	—	—	—

Total	4.26	4.06	2.32	93.9	0.14	57.7	1.07	43.4	0.01	170.5	26.6	96.6*	12.2

Mean values for the amount excreted were significantly different, *P < 0.05.

ND: not detected.
