# Supplementary material for: Formation of Short-Chain Fatty Acids, Excretion of Anthocyanins, and Microbial Diversity in Rats Fed Blackcurrants, Blackberries, and Raspberries
Source: J Nutr Metab. 2013 Jun 24;2013:202534. doi: 10.1155/2013/202534 (PMC3707259; doi:10.1155/2013/202534)
Supplement: Supplementary file 1 — Anthocyanins and anthocyanidins were extracted from blackcurrants and blackberries using a three-step extraction; ethyl acetate extraction, methanol extraction and methanol extraction of hydrolysed anthocyanidins. The extraction for raspberries was a two-step extraction; ethyl acetate extraction and methanol extraction. [file 202534.f1.pptx]

## Slide 1
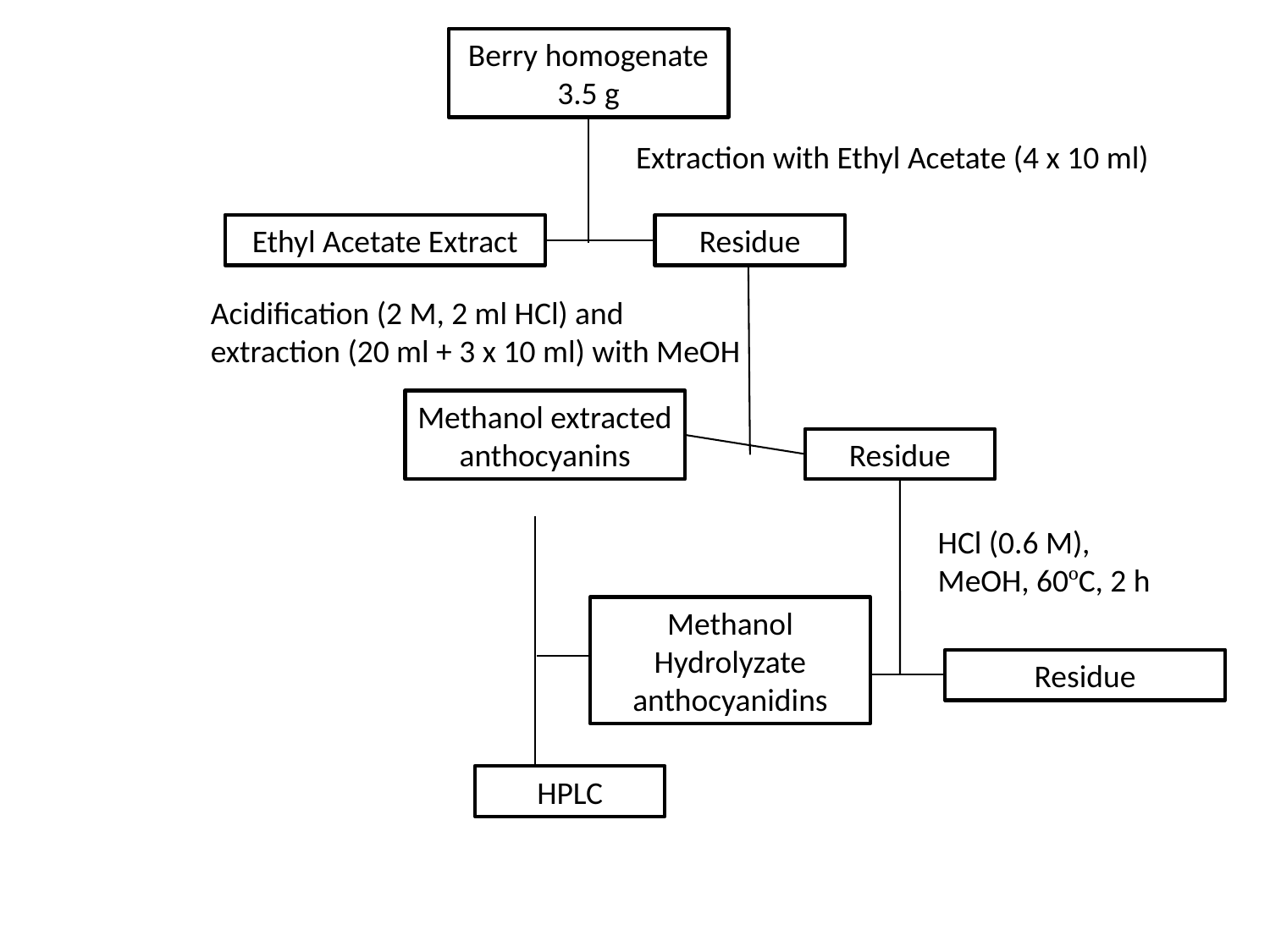

Berry homogenate
3.5 g
Extraction with Ethyl Acetate (4 x 10 ml)
Ethyl Acetate Extract
Residue
Acidification (2 M, 2 ml HCl) and
extraction (20 ml + 3 x 10 ml) with MeOH
Methanol extracted anthocyanins
Residue
HCl (0.6 M),
MeOH, 60ºC, 2 h
Methanol Hydrolyzate anthocyanidins
Residue
HPLC

## Slide 2
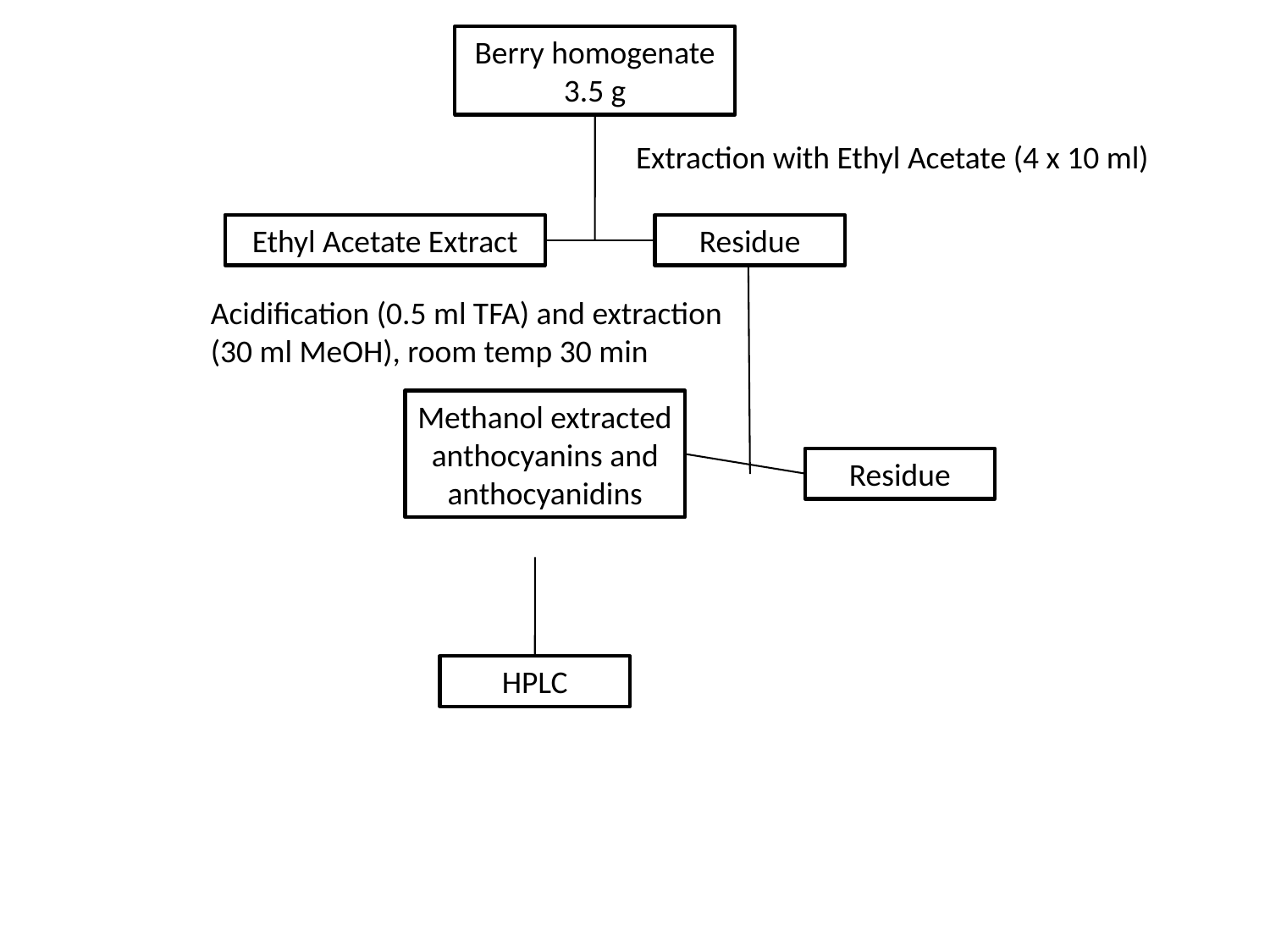

Berry homogenate
3.5 g
Extraction with Ethyl Acetate (4 x 10 ml)
Ethyl Acetate Extract
Residue
Acidification (0.5 ml TFA) and extraction (30 ml MeOH), room temp 30 min
Methanol extracted anthocyanins and anthocyanidins
Residue
HPLC
